# Measuring the association of objective and perceived neighborhood environment with physical activity in older adults: challenges and implications from a systematic review

**DOI:** 10.1186/s12942-020-00243-z

**Published:** 2020-11-09

**Authors:** Manuela Peters, Saskia Muellmann, Lara Christianson, Imke Stalling, Karin Bammann, Carina Drell, Sarah Forberger

**Affiliations:** 1 Leibniz Institute for Prevention Research and Epidemiology–BIPS, Achterstraße 30, 28215 Bremen, Germany; 2 Faculty of Human and Health Sciences, University of Bremen, Bremen, Germany; 3 Institute for Public Health and Nursing Research (IPP), Working Group Epidemiology of Demographic Change, University of Bremen, Bremen, Germany

**Keywords:** Older adults, Neighborhood built environment, Physical activity, Systematic review, Walkability, Objective, Perceived

## Abstract

**Background:**

A supportive environment is a key factor in addressing the issue of health among older adults. There is already sufficient evidence that objective and self-reported measures of the neighborhood environment should be taken into account as crucial components of active aging, as they have been shown to influence physical activity; particularly in people aged 60+. Thus, both could inform policies and practices that promote successful aging in place. An increasing number of studies meanwhile consider these exposures in analyzing their impact on physical activity in the elderly. However, there is a wide variety of definitions, measurements and methodological approaches, which complicates the process of obtaining comparable estimates of the effects and pooled results. The aim of this review was to identify and summarize these differences in order to emphasize methodological implications for future reviews and meta analyzes in this field and, thus, to create a sound basis for synthesized evidence.

**Methods:**

A systematic literature search across eight databases was conducted to identify peer-reviewed articles examining the association of objective and perceived measures of the neighborhood environment and objectively measured or self-reported physical activity in adults aged ≥ 60 years. Two authors independently screened the articles according to predefined eligibility criteria, extracted data, and assessed study quality. A qualitative synthesis of the findings is provided.

**Results:**

Of the 2967 records retrieved, 35 studies met the inclusion criteria. Five categories of methodological approaches, numerous measurement instruments to assess the neighborhood environment and physical activity, as well as several clusters of definitions of neighborhood, were identified.

**Conclusions:**

The strength of evidence of the associations of specific categories of environmental attributes with physical activity varies across measurement types of the outcome and exposures as well as the physical activity domain observed and the operationalization of neighborhood. The latter being of great importance for the targeted age group. In the light of this, future reviews should consider these variations and stratify their summaries according to the different approaches, measures and definitions. Further, underlying mechanisms should be explored.

## Background

Maintaining healthy life years due to lower morbidity through regular physical activity (PA) [1–5] should be a priority as life expectancy increases. Although the benefits of PA are well documented when considering age related diseases [6–8], the prevalence of PA in adults, especially in older adults, in many countries is low [9, 10]. Therefore, it is important to identify modifiable factors that may help older adults to initiate and maintain a physically active lifestyle. Besides individual factors, it is known that characteristics of the neighborhood environment (NE) can affect an individual’s PA behavior [11, 12]. Since mechanisms and features of place and their influence on PA may vary depending on the characteristics of a population group and that group’s sensitivity to certain NE factors [13], research must be specifically focused on the targeted population. Vulnerable groups such as older adults experience the highest risk of inactive lifestyles due to their declining health and reduced functioning and mobility [14, 15] and may thus be most influenced by NE features [16]. Hence, designing age-friendly neighborhoods, which aim to reduce barriers to PA [17] and might make older people feel more confident and secure, calls for the consideration of the effect of the built NE on PA in this age group [18].

### Theoretical and conceptional frameworks for research on the associations of NE and health

Research investigating the associations between the NE and health behaviors comes from multiple disciplines (e.g., public health, urban planning, geography and psychology) [19–25]. Health-related research has mostly focused on individual aspects, such as demographics, health status, psychological, social and lifestyle factors [26–28]. However, some authors have pointed out that environmental variables also need to be considered in health promotion research [25, 29, 30]. In their ecological model, Barton and Grant [31] identified the built NE as a key factor that influences individual behavior and wellbeing. Thus, the socio-ecological framework, which combines the effects of social and physical aspects of both the objective and the perceived NE [25, 30], has been widely used to gain a greater understanding of the relative influence of NE on PA [30, 32, 33]. Since the complex underlying mechanism is also based on different domains of active living [31, 34], which in turn may relate to different NE characteristics [35], the importance of an interactional context and domain-specific approach has been proposed in broader models [36, 37]. To maximise the impact of policy and health interventions, knowledge of interacting factors is necessary. Hence, studies started to address personal perceptions of NE factors such as crime [38, 39] and traffic [38–40], leading to the development of new measures, such as the International Physical Activity Questionnaire (IPAQ) [41], assessing PA for multiple purposes. In addition, it was acknowledged that factors such as attractive and well-designed NEs may help to promote active transportation as well as recreation [42]. Thus, over the last 2 decades a variety of potential factors that may influence PA has been identified, for example, walkable neighborhood designs, access to parks, availability of public transport, and the quality of pedestrian and bicycling infrastructure [43, 44]. In their review in which they looked at walking and cycling in the neighbourhood, Pikora et al. [45] identified a framework with features related to function, personal safety, aesthetics and the presence of destinations. The framework then became an effective international system for classifying built NE characteristics.

### Definition and relevance of objective and subjective measures

Measurement of the built NE can be grouped into four types: (1) self-reported measures [11], (2) systematic observations (e.g. by google street views), community audits [24, 46–48], (3) government statistics [12, 49], and (4) geographic information system (GIS) measures [12]. Several self-reported measures have been evaluated [50] and are typically collected using surveys, interviews or focus groups. The most frequently used tool, which is in line with the conceptual framework by Pikora et al. [45] is the Neighborhood Environment Walkability Scale (NEWS) [11] or the abbreviated version (ANEWS) [51, 52]. The instrument allows for the assessment of multiple dimensions of the perceived NE for PA. Perceptions (e.g. of the quantity and quality of facilities and traffic conditions, safety, barriers) provide data on unobservable psychological processes triggered by satisfaction of the NE. But these may not accurately represent the ‘real’ (objective) environment [53–55] and include the possibility that individuals may in fact perceive the exact same NE context differently [5, 56]. Objective measures include, for example, distances to a specific destination [57], or access to facilities within defined radius of the individual’s home [33]. In order to gather objective data the use of GIS tools in combination with secondary data, such as those provided by government continue to increase through the expanding technical possibilities and their usability. This is now reflected in the growing number of studies [58, 59] conducted using this tools. Features which are not incorporated in such sources can be assessed through direct observations by several audit tools which have been developed in recent years and can complement perceptions of the NE [60, 61]. Objective measures may not reflect the current situation and the needs and experiences of individuals and might eliminate this impact on their PA behavior [54]. On the other hand they can capture important structural aspects of the NE, that may not be perceived [54]. Consequently, studies including both measures have exhibited different and independent associations with PA outcomes in the past [5, 55, 58, 62–70]. Therefore, research that exclusively uses either objective or self-reported measures of NE characteristics may produce limited results to the question of whether perceptions or the objective NE are more reliably associated with PA [54].

### Older residents and their neighborhood: state of science and limitations

It is posited that with increasing age and functional limitations, individuals become more sensitive to environmental barriers (such as large distances, poor quality pavements) and safety issues [71–73]. Associations between NE and PA have been found to be the strongest around retirement age [18], when older adults are more likely to spend most of the time in their neighborhood [74] and thus, are more likely to be affected by their surrounding NE [5, 75]. A neighborhood refers to the geographical area, within which people spend their time, including the physical and social conditions [15]. The scales to be used in research of NE and PA may differ depending on context and purpose [76] and can have different connotations depending on different target groups and individual interpretations of “the neighborhood” [77]. It has been hypothesized that older adults might not walk as far as adults, adolescents and children, who engage in PA through travel to school/work from home during the day and, in addition, are only able to walk within limited geographical areas of their homes [15, 78–80]. Compared with younger adults, older adults tend to be more sedentary and make fewer and shorter trips [81]. Thus, NE characteristics within smaller buffer sizes around home seem to be more applicable to older adults than those typically used for younger age groups [82]. Furthermore, the choice of neighborhood definition may differ for different domains of PA (e.g. for transportation or recreation) and the behavior being studied (e.g., walking, cycling) [37]. Thus, researchers have been using a variety of neighborhood definitions, which also depends on whether exposures are observed within or beyond (e.g. according to activity spaces in terms of daily visited locations) the residential neighborhood [83]. Hence, there is little consensus as to the most appropriate geographic scales [84–86]. This not only weakens the correlation between perceived and objective measures of the NE [37], but can also change the results according to PA [84, 85, 87] and makes it difficult to combine evidence across studies [85]. Consequently, the challenge of defining a neighborhood has been regularly highlighted, though an operational definition has yet to be agreed on [21, 60, 87, 88].

### Review aims

Recently, some studies examined links between the NE and PA in general [58, 89–92], while others investigated adults’ PA [33, 42, 46, 90, 91, 93], and a smaller number focused on children [94, 95]. Although the NE is considered to be especially relevant for older adults, only a few reviews have investigated how NE correlates with PA targeting this particular age group [5, 8, 78, 96–98] and, in addition, have highlighted the difficulties in summarizing evidence across studies. This might be due to methodological limitations including differences in the following: the number, variability and operationalization of measures of NE and PA outcomes [99, 100], the geographical scale, the location of the sample group [15], the choice of measurement and, lastly, the PA domain being analyzed [33, 101, 102]. The aim of this review was hence to assess each of the listed differences. Since the NE can be modified, a body of evidence synthesis based on a proven and comprehensive understanding of the relevant characteristics that act either as facilitators or barriers to PA of older adults is required. This, in turn, has the potential to inform practices on how to develop interventions and environmental designs that underpin active healthy aging [103]. The consideration of both objective and perceived measures of the NE can improve the understanding of how these overlap, interact, and complement each other in reflecting the NE context [11, 12, 70]. In addition, they have proved not to be necessarily in agreement, e.g., perceiving the NE to be less walkable than it really is, which in turn is associated with lower levels of PA [67, 104, 105]. Thus, in contrast to previous reviews, which included studies that used either perceived or objective measurements, or a combination of both to assess NE [5, 96, 97, 99], this review is based exclusively on studies that include both objective and perceived measured data within the same sample.

Since generating evidence based on a synthesis across studies in this field has proven to be difficult, largely due to the differences described above, the aim of this review was to create a sound basis on which future reviews can be prepared.

## Methods

This paper details a review of the literature using both objective and perceived NE features within the same study population regarding PA. Details of the protocol are registered in PROSPERO (Registration number: CRD42018117549). During the extraction and screening of the data, the numerous methods and approaches applied became apparent. This meant that current research methods had to be examined and summarized first, and then a decision made regarding how to summarize them in order to provide evidence for the outcome. Thus, this in-depth synthesis focused on the findings regarding the ways in which objective and perceived NE features were used or linked to investigate their influence on PA.

The review followed the Preferred Reporting Items for Systematic Reviews and Meta-Analyses (PRISMA) statement [106]. The detailed report is attached as Additional file 1.

### Search strategy and information sources

The identification of relevant keywords for the search string was conducted by the author team with the help of a research librarian. Search terms were formulated using synonyms following the strategies used in existing literature reviews that have previously assessed interactions between PA and NE [5, 96, 107], and combined by Boolean operations and MeSH terms and other index terms related to PA as well as perceived and objectively measured NE. The strategy was developed and pretested for MEDLINE in an iterative process and adapted for other databases. The search was conducted in MEDLINE (via Pubmed), Cochrane Central Register of Controlled Trials (CENTRAL, via Cochrane Library), Cumulative Index to Nursing and Allied Health Literature (CINAHL, via EBSCO), GreenFILE (via EBSCO), Physical Education Index (PEI, via ProQuest), PsycINFO (via Ovid), Science Citation Index (SCI) and Social Sciences Citation Index (SSCI), via Web of Science (WoS). An example of the complete search syntax used for MEDLINE is illustrated in Additional file 2. The search strategy was modified for other databases. To identify additional eligible studies and to ensure a comprehensive identification of eligible sources, reference lists of the articles included and previously published systematic reviews and meta-analyses were manually screened for relevant publications. Searches were executed in July 2020. No restrictions on language or publication year were applied.

### Eligibility screening and inclusion criteria

#### Screening strategy

The first author of this review conducted the database search, exported and de-duplicated the results using the reference management software ENDNOTE X9 [108]. The remaining articles were uploaded into RAYYAN QCRI 0.0.1 [109], an online tool for systematic reviews. Two authors independently screened articles in a two-step process: firstly, titles and abstracts were screened to select relevant studies. Secondly, full texts of relevant studies were examined. The screening procedure was based on predefined inclusion and exclusion criteria. The authors discussed any disagreements until consensus was reached. Where consensus could not be reached, a third reviewer was consulted. All reasons for exclusion of full texts were recorded and are provided in Additional file 3. The process of the database search and screening followed the PRISMA-guidelines [106].

#### Inclusion and exclusion criteria

Inclusion and exclusion criteria for the initial systematic review were defined in line with the Population, Intervention/exposure, Comparator, Outcome (PICO) criteria [110]. For this review, all studies were eligible if they met the following criteria: Community-dwelling adults aged ≥ 60 years (or age-stratified analyses in studies with a larger scale) regardless of sex, ethnicity or geographical setting (P). To be included, studies needed to investigate the influence of both objective and perceived attributes of the NE (I and C) on any kind of objectively measured (e.g., by accelerometer, pedometer) or self-reported PA (e.g., by questionnaires or diaries) (O). Studies that (1) examined individuals under 60 years of age or did not provide subgroup analysis of age, (2) investigated specific populations living in nursing homes or other institutional facilities, (3) dealt with either objective or perceived NE measures; and (4) neither examined PA as a primary nor secondary outcome were excluded. All types of peer reviewed empirical studies (i.e., randomized controlled trials, non-randomized controlled trials, cross-sectional, longitudinal, quasi-experimental studies, prospective or retrospective cohort studies, case studies, and qualitative studies) were eligible for inclusion. Non peer reviewed articles, study protocols, books, book chapters, dissertations, commentaries, editorials, and systematic reviews were excluded. The detailed eligibility criteria can be found in Additional file 4.

## Analyses

### Data extraction and organization of the material

The data extraction covers issues such as the types of Studies, types of Data, types of Methods and Outcome (SDMO-criteria), as suggested in the Cochrane Handbook for conducting methodological reviews [111, 112]. The extraction form and categorization scheme to organize the material were developed iteratively during the screening and planning phase and pretested in an iterative process. The extraction form, supported by written instructions on how to record and categorize the data, was provided to all authors involved in the data extraction. A final database of all forms was constructed using a customized Excel sheet containing the following information: first author and year of publication; year of data collection and study design, if applicable, study name; location and setting (e.g. urban/rural); sample characteristics (response rate, sample size, sex, age); neighborhood (definition and strategy for selection of study areas); measures for objective and perceived NE as well as PA (instrument, operationalization and whether it was validated); if applicable, a list of moderators/mediators and a section that describes how objective and perceived data of NE were linked. In the process of data extraction, objective NE data were coded as either (1) taken from commercial or official sources, including administrative/government data, (2) derived by using Geographical Information Systems (GIS), including Open Street Maps (OSM)—data or (3) collected by conducting environmental audits. Perceived NE attributes were organized as observed from respondents, e.g., by using well established and validated instruments such as the NEWS assessment [11, 62], or by requesting perceptions through selected items. In line with previous systematic reviews [5, 96], objective and perceived NE attributes potentially reflecting the same or similar constructs were classified into categories primarily corresponding to the established and most frequently used dimensions from NEWS [11], the most popular instrument for measuring perceived neighborhood environmental attributes worldwide, complemented by additional attributes appearing in the selected articles.

Based on the dimensions of the NEWS/NEWS-A-questionnaire, the attributes were arranged into five NE categories:

1. overall walkability measured by any kind of calculated index or score of perceived or objective data, including information on access to services/land use mix, residential density and/or street connectivity,
2. a residential density/urbanisation category, including connectivity and proximity patterns,
3. a category summarizing factors reflecting the availability and access to services and destinations that have proven to be potentially beneficial for PA, with the subcategories comprising land use mix/diversity, shops/commercial destinations, availability of pedestrian and cycling infrastructure parks/green spaces/recreational destinations,
4. a category aggregating the safety dimension in its expressions of safety from crime, safety from traffic and other security-related aspects such as the presence of street lighting and physical barriers, and lastly a category,
5. which mainly includes qualitative aspects, such as footpath quality, aesthetics, orderliness, littering/vandalism/decay and air pollution and social aspects.

PA measures were categorized into total walking (including total NE walking and walking level) as well as walking for recreation and walking for transport. Likewise, PA was also classified into total PA (including total PA, PA level and MVPA), PA for recreation, and PA for transport. As a final category, sedentary behavior was included, as this might indicate the effect of the determinants in the opposite direction. For this review PA levels were collected as reported in the studies, e.g. in terms of intensity, as moderate-to-vigorous PA (MVPA), energy expenditure, steps per day, time of activity per day or as a verification of compliance with the recommendations for PA.

Since neighborhoods can be operationalized differently, multiple options were created for the extraction form. This includes several radii around the participant’s home for the operationalization of objective neighborhood and default distances between five, 10, 20 or more minutes around the individual’s place of residence, in order to define the perceived NE.

Two reviewers independently extracted all relevant information from included studies, verified each other’s work for accuracy and resolved any discrepancies by taking a similar approach as already outlined. If relevant information was missing from the full-text article, the corresponding author of the study was contacted for clarification. Additional file 5 provides the relevant information of the included studies.

### Assessment of methodological quality

All included studies were assessed for methodological quality by two reviewers using a self-developed checklist adapted from existing sets of criteria used in earlier reviews [5, 99, 113–117], as well as the Quality Assessment Tool for Quantitative Studies (EPHPP) [118]. The criteria were chosen to reflect relevant aspects for a methodological investigation and thus focused on assessing the selection of the sample, measurement and data issues. The eight-item checklist covered whether the study achieved a set of criteria. Two aspects in order to reflect representativeness: (1) stratification of study areas or participant recruitment by key environmental attributes and (2) achievement of adequate participant response rate (≥ 60%) or sample shown to be representative of the population. Further, the evaluation of whether potential confounders were included in the analysis in terms of adjustment for key socio-demographic covariates (at least age, sex, and education) on (3) NE-level and (4) on individual-level. Regarding the data, instruments and analyses, it was assessed whether descriptions of valid or well established measures of (5) objective NE, (6) perceived NE and (7) PA; and (8) an appropriate analytical approach (e.g., p-values and confidence intervals) were provided. Studies for which no clear statement could be made on the respective point or which could not be answered with a definitive [yes] or [no] were labeled as “not applicable” [NA]. As with (e.g.) Cerin et al. [99], the study design was not considered in terms of evaluating the strength of evidence of causality, because the majority of studies included were cross-sectional. The results for the individual studies obtained using the tool are shown in Additional file 6.

## Results

### Study selection

**Fig. 1:**
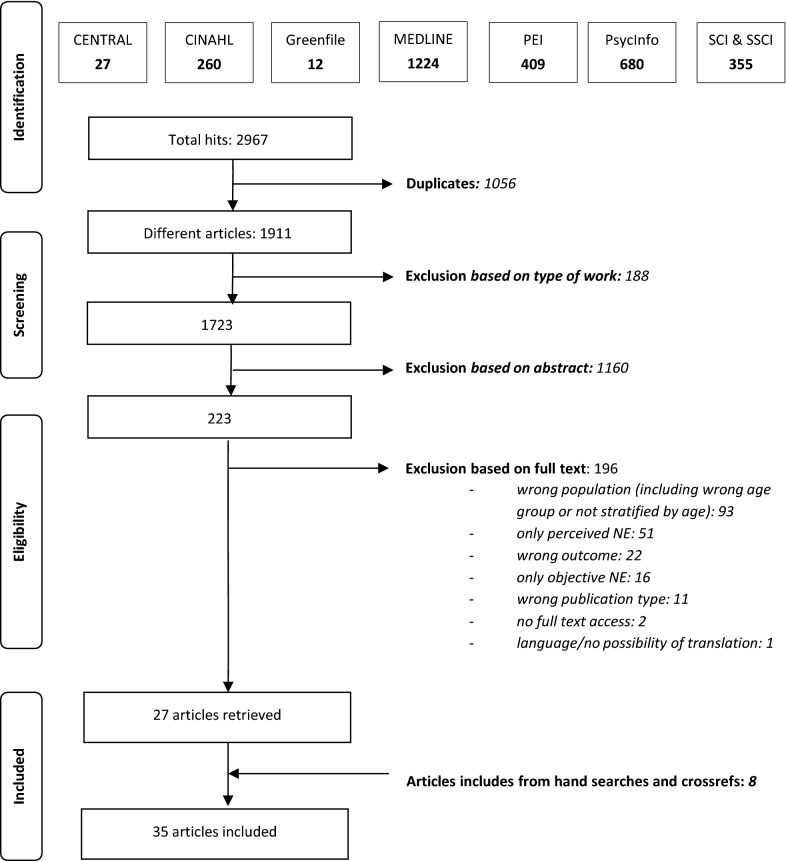
PRISMA flow chart of the screening process and results

The literature search in July 2020 retrieved 2967 articles. After deduplication, 1911 articles were screened following the two-staged screening process. Finally, 35 articles were included in the review (Fig. 1).

### Characteristics, participants and scope of included studies

The characteristics and details of the included studies are provided in Table 1 and, in further detail, in Additional file 5. Among the studies that reported the mean ages of study participants (n = 19), the mean age ranged from 60.5 [119] to 76.9 years [120]. Slightly less than a quarter of the studies (n = 9) included younger participants in their sample, but stratified their analyses according to older age groups; aged at least > 60 years [40, 104, 121–127]. Among the studies that focused on both sexes, the proportion of female participants was, on average, 59.5%, ranging from 36.8% [128] to 79.7% [129]. Some authors focused on women only in their analyses [130–132].

**Table 1:** Characteristics of studies and participants (n = number of individual studies)

Data Collection			
Region	*n*	Year of data collection	*n*
USA	18	2015–2020	1
Canada	3	2010–2015	13
Europe	7	2005–2010	12
Asia	4	< 2005	4
Australia	3	Long-term/cohort-follow-up	2
South America	1	NA	3
Setting		Sample size	
Urban	21	≤ 100	2
Peri-urban (combination of urban and suburban)	2	101–300	7
Suburban	3	301–500	5
Rural	0	501–1000	10
NA	6	1001–2500	5
Study design		> 2500	5
Cross-sectional study	29	NA	1
Longitudinal/observational study	4		
Cluster-randomized intervention trial	2		

*NA* not applicable or not reported

The specific aim to investigate the concordance between objective and perceived neighborhood attributes (or walkability) was formulated by two studies [53, 104]. Fourteen studies examined the relative importance of selected objectively and perceived measured NE attributes on PA in general [120, 122, 123, 129, 133–142] and four studies investigated associations between attributes of the NE and specific PA or walking domains [40, 121, 124, 127]. Some studies aimed to explore the relationships between different NE attributes and PA in a specific population group, for example in adults with type 2 diabetes [119], kidney cancer survivors [128] or overweight and obese urban older adults [143]. One quarter of the studies (n = 9) tested the mediating role of demographic, psychosocial, functional, behavioral, and/or environmental features on the association between NE and PA [125, 127, 128, 130–132, 144–146]. Other studies tested whether associations between NE and PA were moderated by age, sex or education, driving status, crime and safety or objective walkability [82, 121, 126, 147, 148]. The target of one study [149] was to examine the value of PA as a moderating element for somatic health.

### Measurement of PA and NE

**Fig. 2:**
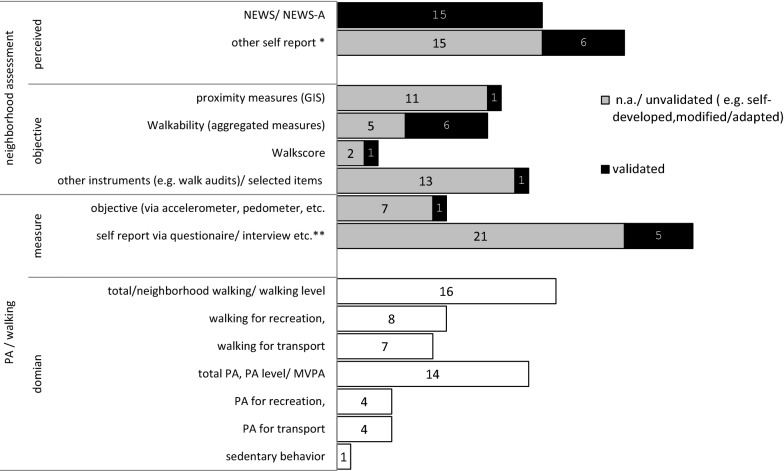
Number and type of assessment of PA and NE within the studies (n = 35). Some studies used more than one option. *e.g., one or more picked single items from existing assessment or self-developed questionnaires/items. **Including IPAQ, short or long (n = 5), EPAQ2 (n = 1), CHAMPS (n = 3), PASE (n = 1), Yale Physical Activity Scale (n = 1), Active Australia Physical Activity Questionnaire (n = 1), NPAQ (n = 1)

Figure 2 summarizes the PA measures and domains, as well as the NE measures aggregated by categories. The exclusive use of self-reports (i.e., by questionnaires, face to face interviews or phone calls) was the most common method for capturing PA (n = 26). Four articles reported that they assessed PA exclusively objectively (i.e., via pedometer, accelerometer) [119, 129, 132, 138]. In five articles [82, 104, 120, 147, 148], both objective and subjective measures were used to assess PA. Nineteen studies used self-generated and/or non-validated instruments to capture PA. Measures of PA included estimated energy expenditure (kcals/day, kcals/week of moderate PA) or total time of PA (mean mins/week of moderate PA). The included studies looked at different characteristics, partly in different PA (or walking) domains as a primary (n = 32) or as a secondary (n = 3) outcome. Fourteen studies reported their defined outcome in more than one domain.

Regarding the assessment of objective NE, the use of GIS derived measures in terms of spatial proximity and/or the calculation of aggregated measures by using geographical and/or governmental databases were the most commonly used approaches (n = 12). Built environment composite indices, such as the validated Walkability Index [150] (n = 6) or modified/adapted forms (n = 5), as well as walk scores, in validated (n = 1) or modified versions (n = 2), were used and typically contained the features density, connectivity and land use mix. The objective NE was measured by more than one instrument in three studies [119, 120, 136]. Perceptions of the environment were usually measured using questionnaires. Among the included articles, 42% (n = 15) used the NEWS assessment [11], 42% (n = 15) measured perceived NE attributes through self-developed instruments, and only a few (n = 6) specified other validated instruments (e.g., walk audits). Two studies [128, 147] combined two instruments: modified scales from NEWS with other validated or self-developed items and scales.

### Definition of neighborhood

Across studies, the definition of neighborhood was inconsistent. With regard to objective operationalization, the variation ranged from (a) administrative specifications (e.g., based on postal code or district level), (b) different radii around each participant’s geocoded home address calculated as buffer sizes, to (c) spatial definitions based on distances from the participant's home to the corresponding point(s) of interest(s) (Table 2). The most frequently used option was the specification of buffer sizes (including circular and network buffer), defining a zone of 250–500 m around each participant’s home (n = 13), followed by definitions via postal codes or census areas (n = 7). Network buffers are especially preferred when examining transport domains within PA. Two studies covered more than one buffer size. Seven studies defined the neighborhood equivalent to administrative areas (census districts or postal codes), and further seven used other spatial definitions such as straight line, Euclidean or network distances in order to quantify the availability or accessibility of particular points of interest. Two studies did not describe their methods. A 10–20 min walk from home was the most commonly reported perceived neighborhood definition (n = 15). Thirteen studies used other definitions, such as requesting the respondents to think of “your neighborhood”. In one study, neighborhood was defined as reachable within a 5 min walk from home. Eight articles did not provide any information on the definition used.

**Table 2:** Neighborhood definitions and PA domains observed

	Objective							Perceived			
	Administrative^a^	Buffer sizes (meter)^b^				Other^c^	N.A	Walk from home		Other^d^	N.A
		≤ 250	250—500	800—1000	> 1000			5 min	10–20 min		
Arvidsson et al. [104]					∆						∆
Bodeker [140]			∆						∆		
Bracy et al. [147]			∆ ○ ●							∆ ○ ●	
Compernolle et al. [121]						○ ● x				○ ● x	
Dadvand et al. [127]			○						○		
Ding et al. [82]			∆ ○ ●								∆ ○ ●
Duncan and Mummery [138]			∆ ○	∆ ○	∆ ○					∆ ○	
Fisher et al. [144]	∆								∆		
Forjuoh et al. [126]							∆			∆	
Gauvin et al. [133]						∆		∆			
Gómez et al. [134]			∆								∆
Hajna et al. [119]			∆						∆		
Hall and Mc Auley [132]				∆ ○ ●							∆ ○ ●
Hanibuchi et al. [123]	○										○
Hu et al. [139]							∆				∆
King [146]						∆ ○ ●			∆ ○ ●		
Lee et al. [151]	∆									∆	
Li et al. [153]			∆						∆		
Mathis et al. [143]	∆									∆	
Michael et al. [53]						∆				∆	
Mowen et al. [40]						∆				∆	
Nagel et al. [137]			∆	∆						∆	
Nathan et al. [152]			∆ ○ ●						∆ ○ ●		
Nathan et al. [120]			∆						∆		
Ng et al. [125]	○ ●								○ ●		
Nyunt et al. [136]			●								●
Orstad et al. [131]					∆				∆		
Piro et al. [149]	∆										∆
Satariano et al. [145]			∆						∆		
Strath et al. [129]		∆								∆	
Towne et al. [122]						∆ ○				∆ ○	
Trinh et al. [128]				∆					∆		
Troped et al. [130]					○ ●				○ ●		
Van Holle et al. [148]	∆ ○ ●								∆ ○ ●		
Wu et al. [124]	∆ ○ ●								∆ ○ ●		
Sum: objective/perceived assessment											
∆ = 32/28 ○ = 16/16 ● = 11/11 x = 1/1	∆ = 6 ○ = 4 ● = 3 x = 0	∆ = 1 ○ = 0 ● = 0 x = 0	∆ = 11 ○ = 5 ● = 4 x = 0	∆ = 4 ○ = 2 ● = 1 x = 0	∆ = 3 ○ = 2 ● = 1 x = 0	∆ = 6 ○ = 3 ● = 2 x = 1	∆ = 2 ○ = 0 ● = 0 x = 0	∆ = 1 ○ = 0 ● = 0 x = 0	∆ = 12 ○ = 7 ● = 6 x = 0	∆ = 10 ○ = 4 ● = 2 ***x = 1***	∆ = 6 ○ = 3 ● = 3 x = 0
Number of different studies (some studies used more than one buffer size)	∑ = 8	∑ = 1	∑ = 13	∑ = 4	∑ = 4	∑ = 6	∑ = 2	∑ = 1	∑ = 15	∑ = 11	∑ = 8

^1^i.e. postal code, census area

^b^e.g. circular or network buffer around participant’s home addresses (Additional file 4 provides the exact definition for each study)

^c^e.g. straight line distances, Euclidian distances or network distances

^d^e.g. “in your neighborhood”

∆: total/neighborhood walking/walking level; total PA, PA level/MVPA

○: walking/PA for recreation

●: walking/PA for transport

x: sedentary behavior

### Observed NE attributes and domains

**Fig. 3:**
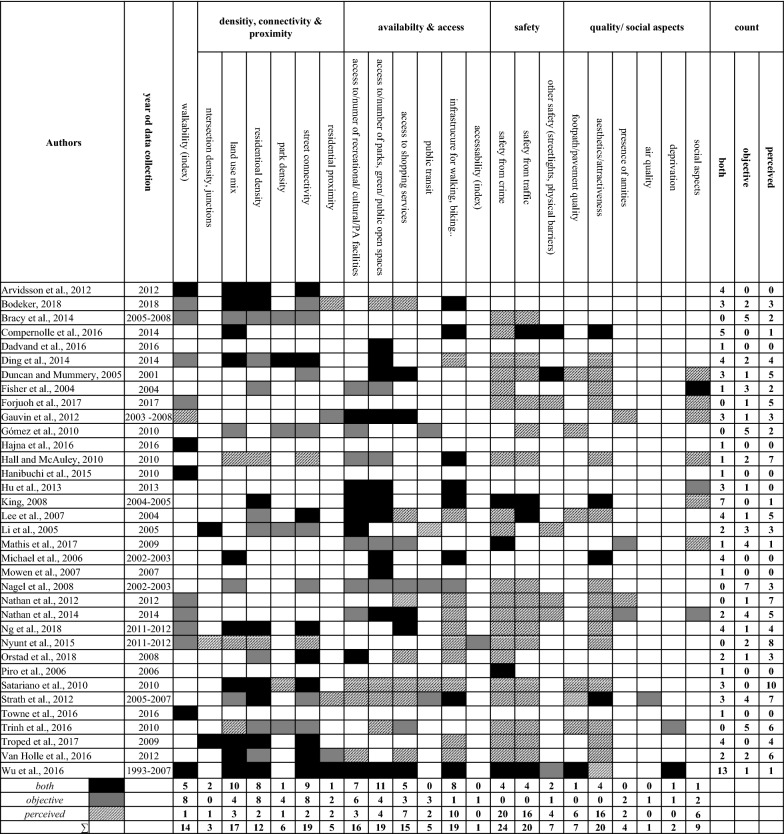
Number of NE attributes tested by the studies (n = 35), sorted by dimensions. Note: Walkability typically contained the features density, connectivity and land use mix

Among the studies included, the association between safety from crime and PA was examined the most (n = 24), with only 17% (n = 4) of these were using objectively collected measures (Fig. 3). Nineteen studies investigated the existence of, or access to an infrastructure for walking or cycling. To measure access to an infrastructure for walking or cycling, eight studies used objective data, ten relied on participants' self-reports and one used both objective and perceived data. Perceived safety from traffic (n = 16) and perceived aesthetics and attractiveness of the NE (n = 16) were recorded in 46% of the studies. One study calculated an objective accessibility index, while another objectively observed air quality. Some studies (n = 6) collected a number of features, using both the objective and the corresponding perceived determinant. Here a pattern can be identified—namely, in the sense that particular attributes were collected via both methods, for example, land use mix (n = 10), street connectivity (n = 9), access to/number of parks, green/public open spaces (n = 11), or the infrastructure for walking/cycling (n = 8). Three studies collected a number of attributes simultaneously [121, 124, 146].

### Synthesis of methodological approaches

With regard to the way in which objective and perceived NE determinants were linked, five different types of methodological approaches were identified (Table 3) as follows:

1. a direct comparison of corresponding objective and perceived NE determinants,
2. an indirect comparison based on particular, though not necessarily equivalent, set of variables reflecting the corresponding NE: objective and perceived,
3. objective or perceived NE attributes used as an interaction term or moderator for each other's effect on PA,
4. modeling purposes: either to define the neighborhood [140] or to stratify perceived NE attributes according to the objectively high or low walkable areas [151, 152], that are determined in this way, and
5. combining a set of objective and perceived determinants to investigate how the different NE factors affect the outcome of the study, without the intention of comparing objective and perceived attributes in a targeted manner.

**Table 3:** Types of methodological approaches

Category		n	References
1	Direct comparison	6	[53, 104, 119, 121, 124, 127]
2	Indirect comparison	7	[122, 123, 126, 128, 138, 139, 149]
3	Interaction/moderation	7	[125, 130, 131, 137, 146–148]
4	Modeling/stratification	3	[140, 151, 152]
5	Combination	12	[40, 82, 120, 129, 132–134, 136, 143–145, 153]

### Observed moderators and mediators

With regard to PA, sociodemographic factors (i.e., sex and age) were the most frequently examined moderators at the individual level. At the NE level, objectively measured walkability [148], a Walkscore [126], buffer sizes [137] and primary type of buildings in the neighborhood [145] were assessed. However, the majority of studies (n = 19) examined self-reported features such as mental health status [127, 131], perceived PA [40, 125, 127], perception of crime and safety [145–147], perceived NE [130, 131], social cohesion/support [127, 132, 144, 146], health/medical condition or functional limitations [132, 149], self-efficacy [132], perceptions of proximity [153] and self-reported park visits [40]. Potential influencing factors for the perception of the NE, such as driving status [82, 145], objective walkability [126, 148], individual-level demographics and income [121, 126, 148], dogs in household [126], as well as body mass index (BMI) [126] were also included as moderator variables.

### Methodological quality of the studies

**Fig. 4:**
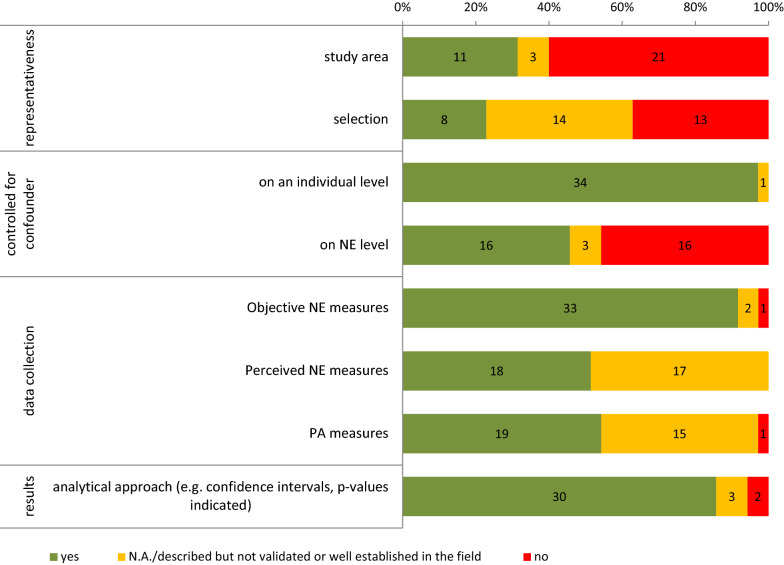
Synthesis of the quality of the included studies (proportion of criteria met, not met, NA)

The methodological quality of the included studies varied (Fig. 4). Among the studies that reported response rates (n = 18), the lowest response rate was 7% [122] and the highest was 71% [140]. Key sociodemographic covariates (i.e., as a minimum age, sex, and education) were adjusted for in nearly all studies (97%) at the individual level. Adjustment at the NE level was done in almost half (46%) of the studies. However, only 31.4% (n = 11) of the articles reported a stratified recruitment sampling according to at least one key environmental attribute, e.g., walkability [104, 129, 145, 147, 148, 151, 152], socio-economic status (SES) or income at the NE-Level [104, 121, 134, 146–148], residential density [121], environmental characteristics (e.g. rural/urban setting), governmental definitions (e.g., counties) [145], age groups [146], violent crime rates [146], or other characteristics [53]. The majority of studies (94%) described the objective NE measures they used as being valid or well established in the field. In contrast, the information regarding measures of perceived NE (51%) and PA (54%) seems to be less valid. Some information on the perceived measures was lacking, measures were insufficiently described, or non-validated measures were used. Of the 35 included studies, 30 (86%) provided appropriate and comprehensible analyses (e.g. indications of confidence intervals and p-values). The results of the methodological quality assessment of the individual studies are shown in Additional file 6.

## Discussion

As the built NE has been the focus of much research over the last decade, a number of studies have provided important insights of how the NE contributes to PA in older adults. Since earlier systematic reviews highlighted how the use of different methods and approaches in primary studies made the summarization of evidence and the performance of traditional meta-analysis with exact quantification of effect sizes difficult, the aim of this review was to identify and summarize these differences to create a sound basis on which future reviews can be prepared. The results of the literature review yielded different approaches, which consequently led to a different selection, combination and operationalization of the NE features included. The first review on this topic, conducted 16 years ago, identified only a few studies exclusively based on self-reporting [78]. Over the last decade there’s been an increase in studies investigating the relationship between NE and PA in older adults-simultaneous with the ongoing development of GIS tools and instruments for the assessment of PA (e.g., by tracking tools). Consequently, all eight studies in this review, where accelerometers have been used, were published within the last 10 years, thus highlighting how accelerometry has become an important technique for research in this field.

Our review revealed a number of NE features investigated for their association with PA, including aspects of safety and aesthetics, typically derived by an individual’s perception and/or environmental audits. However, it has been assumed that individuals who regularly engage in PA in their neighborhood may have more accurate perceptions and be aware of specific characteristics of their NE. This could lead to a misconception concerning cause and effect. In contrast, objective measures (e.g., based on street network data or generated from OSM) may not be optimally operationalized and are prone to inaccuracy and of less actuality [19, 20]. Moreover, objective aspects were often operationalized differently across the studies included in this review and a few of them examined more than one construct. Regarding dimensions of availability and accessibility, the most commonly observed features were land-use mix, distances to common destinations, presence of green and recreational spaces and composite measures (walkability). Since these dimensions indicate a concept that not only covers spatial distances, but also social and material aspects (which tend to differ depending on geographical regions and might lead to different perceptions) [154], there is a need to combine objective factors with others that reflect the cultural and social background, as well as economic conditions of neighborhoods. In this context, our review confirms the distinct North American bias of research already demonstrated in previous reviews [5, 96], which, in addition, took place predominantly in urban areas. Because findings drawn from this can neither be generalized nor transferred to other settings and regions, Frank et al. [150] has already emphasized the importance of conducting similar studies across a wider range of other countries.

Early literature in this field was limited to recreational PA [91], and it was later recognized that PA/walking can also serve for the purpose of transportation [42]. In this review, a number of different PA domains were observed across the studies. Since PA is likely to differ by the PA domain, this might be an obvious explanation for the inconsistency of generalized results. For example, consistent associations of selected NE features have in the past been found for walking for transportation, but not for the recreational domain and total walking and vice versa [1]. Thus, an explicit definition of the outcome with regard to the PA domain studied is of importance, a point that was also raised in previous reviews [92, 99, 155, 156]. Nearly a third of the studies included in this review measured PA via self-reporting. Earlier studies obtained different results depending on whether PA was measured objectively or self-reported [55, 58, 62–69]. Although, specific PA domains can only be measured through self-reports, the validity of such data is to be interpreted with an element of caution as it is susceptible to overestimation of PA [157, 158]. Thus, future reviews and meta-analyses should stratify their analyses with regard to the method of data collection for PA.

The correspondence of the neighborhood in which objective data is collected to the neighborhood to which the perceptions refer, as well as the discrepancy between perceived and objective distances, remains a major methodological challenge in summarizing valid results statements on the subject to be researched, particularly in older adults [55, 159, 160]. Objective definitions of the neighborhood are not necessarily suitable for identifying an older person’s daily activity spaces, and perceptions may in fact be more closely aligned to older adult’s definition of the neighborhood. Thus, walking duration is often used as a proxy for distances [55], while the transportation literature suggests that most individuals are prepared to walk up to 20 min to discretionary destinations [5]. In line with this, a 10–20 min walk from home was the most commonly reported perceived neighborhood definition identified in this review. Nevertheless, self‐reported measures were not intentionally developed to address concordance with objectively defined buffers and therefore, may not be an optimal match because of variations in walking speeds (specially within the targeted older age group) [55]. Results from a previous review [58] indicated stronger associations for analyses with smaller versus larger buffer sizes based on analyses for the overall population. However, the results from this review are not necessarily related to the target group of older adults, as different population subgroups are exposed to different NE attributes in different places, and corresponded to different PA domain during a given day [37, 161]. Given the variability regarding the functional mobility and walking speed, there is a need to improve the definition of the neighborhood scale for the targeted age group. In addition, future reviews should distinguish the studies they include according variable buffers sizes, the measures used (e.g., road network buffers, Euclidean distances) and the setting observed (e.g., urban, rural) [162].

Since the NE determinants, which significantly affect PA, can change over time and are rather susceptible to aspects of validity and residential self-selection [25, 99], this review emphasized the dominance of cross-sectional designs in research in this area. More longitudinal or quasi-experimental studies are needed to help establish stronger causal evidence of selected NE patterns to facilitate PA behaviors, instead of just reflecting neighborhood self-selection [90], which undermines associations [163] and is a major limitation of the evidence to date [91, 97, 107, 155]. Controlling for individual factors during the analysis of longitudinal data might allow for the comparison of personal attitudes and preferences along with residential relocations or NE interventions [164, 165]. Studies using audit tools, which typically measure stable observable features, could provide more information on specific NE characteristics, which might be of particular importance for older adults’ PA.

Lastly, the methodological quality of individual studies should be taken into account when synthesizing evidence. The present review shows that some assessments were only partly reported, insufficiently described, or even based on self-generated, non-validated instruments. Missing information on the levels of significance of the data presents a further challenge. In order to draw meaningful conclusions, confounders, at the individual and NE level need to be considered, and a comprehensible description of all measurements needs to be done. As sampling is a major problem, with many studies reporting very low or no information on response rates, studies should also provide an assessment of the representativeness of the sample and a description of the potential pattern of selection bias [99]. It is particularly important to implement strategies aimed at maximizing the response rate, which have been successfully employed in studies in adults [166].

To gain a broader understanding of NE characteristics and their impacts on multiple levels of PA in old age, novel theories and instruments, based on the growing amount of research, would be desirable [15]. In this regard, new measures of the built NE, such as space syntaxes have recently been explored [167]. A practical basis for consensus in data to be gained from future studies has been provided by the IPEN consortium. This is through standardized templates for creating GIS variables, including operational definitions, examples of computations, documentation of operationalization, and performance of measures in an international context [168, 169]. In addition, new measures such as adaptations of the NEWS tailored for special regions (Sub-Saharan Africa and India) and an internationally applicable instrument for observing streetscape characteristics have been developed [170]. Structured results according to the dimensions covered and features included, enriched by findings from non-North-American as well as rural regions would be of interest. An orientation towards protocols, which have been developed to describe best practices for summarized evidence, would be helpful. However, this would first require that additional individual studies be conducted.

### Strengths and limitations

One of the strengths of this review is that it is the first to systematically summarize the different methodological approaches for analyzing both objectively and perceived measured NE, in relation to PA. Furthermore, the analyses focused on older people, as there are indications that they may be most influenced by NE features and have special requirements for the NE in order to be physically active [171, 172]. Only few authors of earlier reviews focused on older adults in this context, and only one examined studies that exclusively included both objective and subjective measures of NE, but only focused on the Australian territory [89]. Thus, although knowledge about these relationships is becoming increasingly important for future health care policies, this relevant population has been understudied to date. This review however also has several limitations. First, the review was comprehensive but not exhaustive, meaning that all work that was previously conducted is not necessarily included. This might be a consequence of the limitation regarding the selection of data sources for the search or the decision to omit grey literature. For instance, no searches were done using google scholar. In addition, there was no access to the EMBASE database, which may have limited the results. Second, during the literature review we did not explicitly search for single PA domains according to special NE attributes (e.g. PA/walking for recreation linked with exposure of green spaces). Hence, future reviews should clearly define the targeted PA behavior and environmental exposure in order to obtain an extensive overview. Because the theoretical framework on which the selection of the respective indicators within the included studies is based remains unknown in most cases, the underlying concepts can only be assumed. Thus, the process of data extraction and the categories created were organized on the basis of a personal assumption of a conceptual framework, in order to achieve an optimal comparison. For some issues, such as the classification of the NE attributes investigated, the outcome was aggregated to a general meaning of PA, although special PA domains were observed. This contributed towards simplifying the description of the findings.

The quality assessment was challenging because traditional quality assessment tools were found to be not suitable to evaluate studies in the context of NE and PA [89, 115]. Hence, a quality checklist adapted from earlier reviews in this field was used. The checklist comprised relatively simple criteria, more suited to the context. Regarding the evaluation within this review, an assessment of residential self-selection is lacking, as this is rarely undertaken in the included studies.

## Conclusions

As the condition of the NE is affected by policies across multiple fields, such as transportation, planning and public health, evidence on the impact and the relation of both, objective and perceived NE characteristics on PA among older adults could guide interventions to promote PA tailored for the specific needs of the older age group. In order to obtain robust evidence that could stimulate changes, compatible and comparable methods and reporting standards are of utmost importance. Since associations with PA vary according to the different measurements of PA and NE as well as the PA domain observed, future reviews should consider these differences when conducting literature searches, and should define eligibility criteria more precisely based on this knowledge. In addition, the use of standardized, reliable and validated measures, based on uniform instruments, suitable for the group of older adults should be encouraged. Further, the harmonization of definitions and reporting standards as well as validated, age-appropriate (objective and perceived) geographic scales could improve the comparability of findings. This will allow for the pooling of data and will help to establish more robust evidence in this field. In the absence of common definitions, future reviews could stratify their summaries pertaining to the differences. Finally, synthesized evidence on the association between NE and PA in older adults based on uniform principles and methods remains scarce.

## Supplementary information

**Additional file 1.** PRISMA-protocol.

**Additional file 2.** Medline search.

**Additional file 3.** Reasons for exclusions.

**Additional file 4.** Eligibility criteria.

**Additional file 5.** Extraction table.

**Additional file 6.** Results of the methodological quality assessment of the individual studies.

## Data Availability

Data sharing is not applicable to this article as no datasets were generated or analyzed during the study.

## Abbreviations

BEPAS: Belgian Environmental Physical Activity Study
BMI: Body mass index
CENTRAL: Cochrane Central Register of Controlled Trials
CHAMPS: Child Health and Mortality Prevention Surveillance
CINAHL: Cumulative Index to Nursing & Allied Health Literature
EMBASE: Excerpta Medica database
ENVINT: Environment international
EPAQ: Electronic Personal Assessment Questionnaire
EPIC: European Prospective Investigation into Cancer and Nutrition
GIS: Geographic Information System
MVPA: Moderate-to-vigorous physical activity
NE: Neighborhood environment
NEWS: Neighborhood Environment Walkability Scale
NEWS-A: Neighborhood Environment Walkability Scale-abbreviated version
NPAQ: Neighborhood Physical Activity Questionnaire
IPAQ: International Physical Activity Questionnaire
OSM: Open Street Maps
PA: Physical activity
PEI: Physical Education Index
PASE: Physical activity scale for seniors
PRISMA-P: Preferred Reporting Items for Systematic Reviews and Meta-Analyses Protocols
SCI: Science Citation Index
SSCI: Social Science Citation Index
SHAPE: Survey of the Health of All the Population and Environment
SNAP: Supplemental Nutrition Assistance Program
SNQL: Neighborhood Quality of Life Study for Seniors
SPOTLIGHT: Sustainable prevention of obesity through integrated strategies
WOS: Web of Science
WHO: World Health Organization
