# Supplementary material for: Measuring the association of objective and perceived neighborhood environment with physical activity in older adults: challenges and implications from a systematic review
Source: Int J Health Geogr. 2020 Nov 9;19:47. doi: 10.1186/s12942-020-00243-z (PMC7654613; doi:10.1186/s12942-020-00243-z)
Supplement: Supplementary file 2 — Additional file 2. Medline search. [file 12942_2020_243_MOESM2_ESM.docx]

**Additional file 2: Medline search (via PubMed on 6^th^ January 2019, updated on 29^h^ July 2020**

| Search line | Terms | Hits |  |
| --- | --- | --- | --- |
| 1* | "physical activity" OR "physical activities" OR sport OR sports OR sporting OR exercis* OR walk* OR bicycle OR bicycles OR bicycling OR biking OR cycle OR cycles OR cycled OR cycling OR "active transport*" OR "physically active" OR pedestrian* | 1,342,151 |  |
| 2 | "Exercise"[Mesh] OR "Sports"[Mesh] | 255,681 |  |
| 3 | "physical environment*" OR walkability OR "objective environment*" OR walkable OR "natural environment*" OR "built environment*" OR "urban environment*" OR "community environment*" OR "neighborhood environment*" OR "neighbourhood environment*" OR "neighborhood design*" OR "neighbourhood design*" OR "built neighborhood*" OR "built neighbourhood*" OR "characteristics of the neighborhood*" OR "characteristics of the neighbourhood*" | 58,585 |  |
| 4 | "Residence Characteristics"[Mesh] OR "Environment Design"[Mesh] | 62,514 |  |
| 5 | "perceived neighborhood" OR "perceived neighbourhood" OR "perceived neighborhoods" OR "perceived neighbourhoods" OR "neighborhood perception" OR "neighbourhood perception" OR "neighborhood perceptions" OR "neighbourhood perceptions" OR "subjective neighborhood" OR "subjective neighbourhood" OR "subjective neighborhoods" OR "subjective neighbourhoods" OR "subjective environment" OR "subjective environments" | 11,168 |  |
| 6 | “Perception"[Mesh] | 400,745 |  |
| 7 | #1 OR #2 | 1,343,984 |  |
| 8 | #3 OR #4 | 86,776 |  |
| 9 | #5 OR #6 | 409,079 |  |
| 10 | #7 AND #8 AND #9 | **905** | **6^th^ January 2019** |
|  | additional hits generated by the search update | **318** | **29^h^ July 2020** |

***Note: **** *Since we focus on types of activities, which have been established in this research area and that can also be measured objectively, for example with an accelerometer or pedometer, the search is limited to certain terms.* *In addition,* *the MESH search might also cover other potentially relevant activities, such as “running”*
