# Supplementary material for: Measuring the association of objective and perceived neighborhood environment with physical activity in older adults: challenges and implications from a systematic review
Source: Int J Health Geogr. 2020 Nov 9;19:47. doi: 10.1186/s12942-020-00243-z (PMC7654613; doi:10.1186/s12942-020-00243-z)
Supplement: Supplementary file 4 — Additional file 4. Eligibility criteria. [file 12942_2020_243_MOESM4_ESM.docx]

**Additional file 4: Eligibility criteria**

|  | Inclusion | Exclusion |
| --- | --- | --- |
| Participants | - Participants aged ≥60 years - Humans - Defined by study as “community-dwelling” or living independently | - Studies only focusing on participants aged <60 years - Non-human studies - Individuals hospitalized long-term, living in long-term care facilities or nursing homes |
| Intervention/ Exposure  (and Comparator) | - Articles that indicate at least one NE characteristic, including walkability, connectivity, safety etc. - Articles that deal with both objective and perceived measures | - Articles that deal with either objective or subjective measures - Articles that neither address objective nor subjective measures |
| Outcome | - PA as primary or secondary outcome - Levels or changes in frequency or amount of participation in PA assessed by objective (e.g., pedometer, accelerometer) or subjective measures (e.g., PA diaries, questionnaires) - PA expressed as any kind of PA or walking or sports domain, exercise or sedentary behavior (as antagonist) - Walking for recreation or exercise, or walking to reach a destination (active travel, non-motorized travel, transport-related PA, destination-oriented walking, and utilitarian walking | - PA is neither measured objectively nor subjectively |
| Type of Publication | - Randomized controlled trials, non-randomized controlled trials, cross-sectional, longitudinal, quasi-experimental studies, prospective or retrospective cohort studies, case studies, or qualitative studies | - Study protocols, books or book chapters, dissertations, commentaries, editorials, or systematic reviews |

***Abbreviations:*** *NE: neighborhood environment, PA: physical activity*
