# Supplementary material for: Measuring the association of objective and perceived neighborhood environment with physical activity in older adults: challenges and implications from a systematic review
Source: Int J Health Geogr. 2020 Nov 9;19:47. doi: 10.1186/s12942-020-00243-z (PMC7654613; doi:10.1186/s12942-020-00243-z)
Supplement: Supplementary file 5 — Additional file 5. Extraction table. [file 12942_2020_243_MOESM5_ESM.docx]

**Additional file 5: Study characteristics of included studies categorized by analytical approach**

| **Author (year),**  **Superordinate/ individual study (year of data collection), country, study design** | **Sample (n), mean age, % female, response rate, stratification** | **Objective NE:**  Neighborhood definition, assessment, factors | **Perceived NE:**  Neighborhood definition, assessment, factors | **PA Outcomes:**  assessment, condition studied, moderators/mediators | **Methodological linkage of**  **objective and perceived**  **characteristics** | |
| --- | --- | --- | --- | --- | --- | --- |
| **approach: direct comparison** | | | | | |  |
| ***Arvidsson et al., 2012*** (1);  SNAP-study (2008-2009);  Stockholm, Sweden;  cross-sectional | n=1925  (≥ 50 ≥ 60= 680);    54.1% females;  30.5% response;  Stratification: Walkability (high/low), Income at neighborhood level); | 1000m circular buffer zone. *;  GIS-based walkability index;  walkability index = z-score of residential  density + z-score of land use mix + 1.5 z-score of street connectivity; | 10 min walk from home^∆;^  self-reported via NEWS;  perceived residential density, land use mix, and street connectivity; | *self-report* via IPAQ long + *objective* assessment via accelerometer;  domain walking (walking for transportation, walking for leisure), total physical activity and moderate-to-vigorous physical activity (MVPA); |  | *Reported kappa statistics for direct comparison of objective and perceived walkability, land use mix, e.g. flats in the neighborhood,*  *residential density and street connectivity.*  *Based on this, the authors examined whether this non-concordance was reflected in PA* |
| ***Compernolle et al., 2016*** (2);  SPOTLIGHT-Study (2014),  Cities & suburbs of Ghent, Paris, Budapest, Randstad, London  cross-sectional | n=5205  Age 18-109, mean 52.2; SD 16.3;  55.3% females;  10.8% response;  Stratification: residential density & SES at neighborhood level | street segments (part of the street between 2 intersections with a min. length of 50 m & a max. length of 300 m);  SPOTLIGHT virtual audit tool (S-VAT) with Google Street View;  walking , cycling , public transport , aesthetics ), land use mix (, grocery stores , food outlets and recreational facility-related items; | online survey & questionnaires (ALPHA & MESA);  presence of facilities (supermarkets, shops, restaurants/bars/cafés, areas for recreation & leisure-time PA); | self-report via Marshall sitting questionnaire (sedentary behaviour); IPAQ long (PA);  domain-specific sedentary time, Moderate-to vigorous leisure-time & transport-related PA in the last seven days;  individual-level socio-demographic variables |  | *Compared association between objective vs perceived environmental and domain-specific sedentary behaviors* |
| ***Dadvand et al., 2016*** (3);  Barcelona Health Survey (2011);  Barcelona, Spain, urban;  cross-sectional | n = 3461 (≥18; ≥ 65 =851);  age 18 - ≥ 65;  52 % females;  43 % response, | Different circular buffer sizes: 100m, 250m, and 500m;  GIS-based analyses based on normalized difference vegetation index (NDVI) & government data;  residential surrounding greenness, residential proximity to green spaces (300m-yes/no) | 10 min walk from home;  self-reported via one item question;  perceived proximity to green spaces | self-report via IPAQ short;  level of PA (moderate/ high);  mediators: mental health status, perceived social support; |  | *Reported kappa statistics for direct comparison of objective and perceived proximity to green spaces. Based on this, the authors examined whether this non-concordance was reflected in PA* |
| ***Hajna et al., 2016*** (4);  (06/ 2006 – 05/2008);  Montreal, QC, Canada, urban;  observational | N= 131;  age: mean 60.5, SD 10.4;  48.1 % females; | 500-meter street network buffers around the centroid of each participant’s home postal code address;  GIS-derived walkability, Audit-assessed walkability, Walk Score;  walkability: street connectivity, residential density & land use mix, walk score: proximity to 13 walkable destinations (e.g., stores) | 10 min walk from home;  self-reported walkability;  presence/ condition of sidewalks, street lighting, traffic, proximity to stores & transit stops, presence of interesting sights, activity level of neighbors, safety while walking | Objective via pedometers;  Count of daily steps; |  | *Reported correlation coefficient for comparison of different measures: perceived/GIS-derived measures; perceived measures/ Walkscore* |
| ***Michael et al., 2006*** (5);  SHAPE (2002/2003);  Portland, OR, USA, urban;  cross-sectional | n = 105;  age: mean 75.1, SD 6.29;  67 % females;  30.5 % response,  Stratification: Walkability: (high/ medium-high/ medium-low/ low | Street level;  GIS-based analyses based on Regional Land Information System (RLIS) + Audit instrument;  sidewalk quality, aesthetics, presence of shopping malls, parks, trails, number of ‘sufficiently active’ people, number of registered dogs, street lighting | “near home”;  face-to face interviews in the SHAPE Survey;  sidewalk quality, neighborhood graffiti & vandalism (aesthetics), presence of shopping malls, parks, trails | self-report via SHAPE questionnaire;  Neighborhood walking level (high/low); |  | *Reported kappa statistics for all corresponding measures.* |
| ***Wu et al., 2016*** (6);  EPIC-Study  (1993, 1997, 2006/2007);  Norfolk, UK, rural & urban;  cross-sectional | n = 8281;  age: urban= mean 67.8, SD 8.4, rural = mean 66.4, SD 8.3;  Urban: 67.4% & rural: 56 % females;  43 % response;  Stratification: urban/ rural | postcodes;  herfindahl hirschmann index to estimate land diversity, GIS-based measures of walkability;  presence of green spaces, land use mix, densities, safety, crime, pavement, streetlights; | 10 min walk from home;  self-reported via adapted NEWS-questions;  access to services, street connectivity, walking & cycling facilities, aesthetics, pedestrian & traffic safety, detached houses in neighborhood | self-report via EPAQ2;    domain specific activity (MVPA)  mediators: mental health status, perceived social support |  | *Comparison of similar objective and perceived characteristics: with regard to selected PA domains.* |
| **approach: indirect comparison** | | | | | |  |
| ***Duncan & Mummery, 2005*** (7);  (08/2001-09/2001);  Rockhampton, Queensland, Australia  cross-sectional | n =1281;  46.6 % response; | radii of 0.5, 0.8, 1.0 and 1.5 km around residential location  GIS-based measures;  Euclidian- & street network distances to the nearest parkland, shopping center, pathway network, busy street (>=60kph speed limit), newsagent; number of active people; number of dogs; roadway within 20m of street light; | N.A.  self-reported via 11 Item-questionnaire;  safety, aesthetics, accessibility, opportunities for PA | self-report via Active Australia Physical Activity Questionnaire;    meeting PA recommendations (>=150 min of PA/week: yes/no); participation in recreational walking (yes/no) |  | *Objective and perceived NE characteristics were analyzed according to their particular influence.* |
| ***Forjuoh et al., 2017*** (8);  (10/2013-06/2014);  Temple, Killeen, College Station & Bryan, Texas, USA, peri-urban (urban and rural features coexist within cities)  cross-sectional | n = 253;  age ≥ 50  46.4 % females;  6.8 % adjusted response; | Walk Score;  accessibility-based measures to various walkable destinations from home, estimate of walkability | self-reported (unvalidated);  neighborhood safety from traffic, from crime, from physical injury risks, behavioral factors in neighborhood safety, social aspects of the neighborhood, attractiveness of the neighborhood | self-report via combination of two survey items;    walking days for any purpose in a typical week, walking minutes for any purpose in each day;  moderators: overweight, social interaction (dogs in household), demographics, walk score |  | *Objective and perceived NE characteristics were analyzed according to their particular influence.* |
| ***Hanibuchi et al., 2015*** (9);  Japanese General Social Surveys (2010);  Japan;  cross-sectional secondary analysis | n = 2395;  age 20-89 (983 ≥ 60 ≥ 89)  53.5 % females;  62.1 % response; | small area codes comparable to a U.S. census-block group;  GIS-based walkability index;  population density, road density, access to parks, access to retail areas, neighborhood deprivation | *N.A.*  self-reported via questionnaire based on JGSS-2010, plus questions about "perceived NE pollution” & “perceived NE sociability;  walkability based on 6 statements | self-report;    leisure-time PA: e.g. exercise, play, sports (walking, swimming, baseball)  mediators: mental health status, perceived social support |  | *Objective and perceived NE characteristics were analyzed according to their particular influence.* |
| ***Hu et al., 2013*** (10);  (2010);  Tainan, Taiwan, urban;  longitudinal, observational study | n = 804 (60 ≥-69 =77)  55.9 % females; | near participant’s home  community audits;  physical environments: parks, green spaces, schools playgrounds, sports fields, gyms, stadiums, bikeways, sidewalks; social environments related to PA: e.g. exercise groups, sports clubs, dancing/aerobic groups, volunteer organizations | near participant’s home;  self-reported via telephone interview;  facilities appropriate for PA near home: e.g. parks, school playgrounds, community activity centers, stadiums or arenas, gyms or swimming pools, open spaces or squares, pavement | self-report via telephone interview;    domain specific activity (MVPA)  domain walking, jogging, sports, dancing, and others (swimming, bicycling, mountain climbing), levels of PA: regular, irregular, no PA in the past month |  | *Objective and perceived NE characteristics were analyzed according to their particular influence.* |
| ***Piro et al., 2006*** (11);  Oslo Health Study  (2006);  Oslo, Norway, urban;  cross-sectional | n = 3499;  age: 75/76 years  50 % females;  58.2 % response; | administrative boroughs;  analyses of administrative NE-level-data  ;  registered neighborhood violence | participant’s neighborhood  self-reported via item-questions;  perceived safety | self-report;    level of PA: less than one hour a week/ more than one hour a week  mediators: health/medical condition |  | *Objective and perceived NE characteristics were analyzed according to their particular influence.* |
| ***Towne et al., 2016*** (12);  (2016);  Temple, Killeen, College Station, & Bryan, Texas, USA, peri-urban  cross-sectional | n = 344;  age: 50 ≥ 64 =171, 65+ =173;  53.8 % females;  6.8 % response;  Stratification: Walk Score | distance from residents address to routine destinations;  Walkscore  score of walkability based on distance to routine destinations (e.g., grocery stores, schools, restaurants, parks, drug stores), higher weights to those located closer and lower to those further away | N.A.  self-reported via questionnaire;  score (high/low):presence sidewalks or protected walkways, - see and speak to other people while walking, many people walk or bike, clean and well maintained streets & walkways , safety. | self-report;    average minutes of walking per week in special domains: walking for recreation, walking for any purpose, grouped into >150 minutes/week and below 150 minutes/week ("meeting recommendation"); |  | *Objective and perceived NE characteristics were analyzed according to their particular influence.* |
| ***Trinh et al., 2016*** (13);  (2010);  Province of Alberta, Canada, urban & rural  cross-sectional | n = 432;  age: mean 64.4; SD 11.1;  36.8 % females;  42.5 % response; | Network buffers of 1 km from the participants’ home postal code centroid  GIS-based measures;  mean household income, population density, park density, number of shopping centers, number of recreation centers, local road density, and intersection density | self-reported via combination of items from NEWS and IPAPSEM - Survey;  perceived proximity to retail shops & recreation facilities, aesthetics, crime, traffic, walking infrastructure quality | self-report via leisure score index (LSI) from the Godin Leisure-Time Exercise Questionnaire (GLTEQ)    meeting PA guidelines (yes/no) |  | *Objective and perceived NE characteristics were analyzed according to their particular influence.*  *.* |
| **approach: interaction/moderation** | | | | | |  |
| ***Bracy et al., 2014*** (14);  SNQLS (2005-2008);  Baltimore, Maryland-Washington, DC & Seattle-King County, Washington, USA  cross-sectional | n = 718;  age: mean 74.4, SD 6.3;  53.1 % females;  Stratification: walkability & neighborhood income: high, low | 500-m street network-buffer around each participant’s home  GIS-based walkability index  walkability index= z-scores of residential density, retail floor area ratio, intersection density, and land use mix | *10min walk from home^∆;^*  self-reported via selected items/ scales from NEWS;  perceived crime, traffic, and pedestrian safety | *self-report* via CHAMPS-questionnaire & *objective* via accelerometer (ACTi Graph)    total physical activity, transportation and leisure walking (MVPA)  moderator/mediator: perceptions of crime and safety |  | *relationship between objective NE and PA were analyzed with regard to a potentially moderating/mediating effect* |
| ***King, 2008*** (15);  Neighborhoods and Senior Health (NASH)  (2004-2005);  Denver, Colorado, urban;  cross-sectional | n = 190;  age: mean 74.2, SD 5.8;  57 % females;  23 % response;  Stratification: high walk, low walk; high crime, low crime; high income, low income; high elderly, low elderly. | census tract  Audit, combination of SPACES and NBOT+ 20 new items;  biking for transportation, children and play areas, detailed checklists of observed illicit activity, types of litter, presence and condition of transit stops, signage indicating senior services or discounts, curb cuts, traffic, crime safety, driving behavior, neighborhood watch sign, dogs, walkability; | *10min walk from home^∆;^*  self-reported via  modified NEWS;  ;  neighborhood walkability, access to resources, traffic, crime safety, neighborhood social cohesion | self-report via CHAMPS-questionnaire;    frequency and duration for each activity for daily living;  mediators: social cohesion, safe from crime, individual perception |  | *interactions between individual perceptions and objective NE were observed* |
| ***Nagel et al., 2008*** (16);  SHAPE (2002/2003);  fifty-six neighborhoods in Portland, Oregon, USA  cluster-randomized intervention trial | n = 546;  Age: mean 74.5; SD 6.3  70 % females;  30.5 % response;  Stratification: leader-guided NE-walking condition vs education-only control condition | municipally defined  GIS-based measures within either the quarter-mile or half-mile buffer  measures of automobile traffic volume on local streets, sidewalk coverage, intersection frequency, and public transportation access, distance from each participant’s residence to the nearest park/green space | face-to-face interviews via SHAPE Survey; follow up questionnaires by phone or mail;  walkability score : gangs, graffiti, violent crime, vandalism, burglary, abandoned or boarded-up buildings, alcohol or drug in the neighborhood, walking safety | self-reported via Yale Physical Activity Scale    Total weekly walking time  miles around participants’ homes |  | *objective and perceived NE (as NE-level covariate) characteristics were according to their potential influences on perceived NE* |
| ***Ng et al., 2018*** (17);  Singapore Longitudinal Ageing Study  (2011/2012);  18 neighborhoods in South Central region in Singapore, urban  cross-sectional | n = 402;  age: mean 69.2, SD 8.44  60.9 % females;  67 % response; | administrative area  GIS-based: Neighborhood Environment for Active Transport (NEAT measurement protocol), Walkability Index  residential density, street connectivity, and land use mix for 18 study area units | *10min walk from home^∆;^*  self-reported via locally adapted NEWS-Scale;  residential density, street connectivity, land use mix – diversity, land use mix – access, infrastructure for walking or cycling, aesthetics, traffic and crime safety | self-report;    PA for Transportation, PA for leisure (frequencies)  transportation PA (TPA) & leisure time (physical, social and productive) activities (LTA) |  | PA as a potential mediator for the effect of NE on RBANS (cognitive functions*) was observed.* |
| ***Orstad et al., 2018*** (18)  US Nurses’ Health Study  (2008);  USA  cross-sectional | n = 432;  age: mean 73.1; SD 6.7  100 % females; | 1200m network buffers around each participant’s home  GIS-based measures;  mean household income, population density, park density, number of shopping centers, number of recreation centers, local road density, and intersection density; | *10min walk from home^∆;^*  self-reported via PANES;  presence of free or low cost recreation facilities, shops, stores, & markets, sidewalks on most streets, crime rate, | self-report; leisure score index (LSI) from the Godin Leisure-Time Exercise Questionnaire (GLTEQ)    meeting PA guidelines (yes/no) |  | *perceived NE as a potential mediator for the associations between objective walkability and PA outcomes was observed.* |
| ***Troped et al., 2017*** (19);  mailing in 2009, based on prospective cohort study started in 1976  Massachusetts, Pennsylvania, and California (USA), urban  cross-sectional | n = 2732;  age: mean 72.8, SD 6.8  100 % females;  Stratification: different cities | 1200m line-based network buffer around each participant’s home  GIS-based measures;  density land use mix, street connectivity | *10min walk from home^∆;^*  self-reported via  modified ANEWS:  land use mix (access to destinations), street connectivity, infrastructure for walking , aesthetics, traffic safety , and personal safety | self-report; using items from CHAMPS survey;  walking for leisure, walking for utilitarian purposes in minutes/week  Mediators: perceived NE variables | . | *perceived NE as potential mediator for the associations between objective NE variables and walking for leisure and utilitarian purposes was observed. Objective built environment characteristics as potential influence for environmental perceptions and walking was observed.* |
| ***Van Holle et al., 2016*** (20);  BEPAS Seniors  (10/2012 - 09/2012)  20 neighborhoods located in Ghent, Belgium, urban & suburban  cross-sectional | n = 438;  age: ≥ 65  54 % females;  40 % response;  Stratification: Walkability (high/low), median household income (high/low) | 1km network buffers from participants’ home postal code centroid  GIS-based walkability Index;  residential density, street connectivity, land use mix diversity | *10min walk from home^∆;^*  self-reported via NEWS;  land use mix diversity; access to recreational facilities; access to services; connectivity of the street network; physical barriers to walking; infrastructure for walking; aesthetics; safety from crime and from traffic | *self-reported* PA levels (IPAQ), validated) + o*bjectively* assessed with ACTi Graph GT3X (+) accelerometers    (MVPA)/PA-Levels,  moderator: objective walkability, income |  | *objective NE walkability was observed as potential moderator for the association between physical functioning and transport walking in general.* |
| **approach: modeling** | | | | | |  |
| ***Bodeker, 2018*** (21);  senior housings estates in Bielefeld, Germany, urban+ rural suburbs  cross-sectional | n = 65;  age: ≥ 65  58 % females;  71.1% response; | 400m network buffer around each participant’s home  GIS-based Walkability Index; GIS procedures for mapping the perceived data  household density, connectivity, land use mix, retail floor area ratio (for Walkability Score), Street and path network, access values for pedestrians | Self-reported (mental mapping interviews);  destinations, they usually walk to, access to services, access to green spaces, public transport | self-reported via modified Neighborhood Physical Activity Questionnaire – NPAQ;    neighborhood walking & total walking (min/day) |  | *NE attributes and their associations to PA according to definitions of neighborhood were observed.* |
| ***Lee et al., 2007*** (22)  2004;  Metropolitan Tokyo, urban + a city from the rural northeastern region, Japan  cross-sectional | n = 772 (high walkable areas: 495, low walkable areas: 277)  age: high walkable areas = mean 62.6, SD 7.6, low walkable areas= mean 60.4, SD 9,5  high walkable area: 54 % female, low walkable areas: 70 % female  Stratification: walkability: high/ low | administrative units  GIS-based measures  residential density and mixed land use, neighborhood street connectivity | Self-reported  accessibility, safety, convenience, aesthetics | self-reported via  self-administered questionnaire  neighborhood walking & total walking (min/day) |  | *objective determinants were mainly used to model the NE or Walkability (high walkable/ low walkable areas). Not for a detailed comparison.* |
| ***Nathan et al., 2012***; (23)  2012  92 villages in the Perth metropolitan and Peel regions, Australia  cross-sectional | n = 325;  age: mean 76,9, SD 7,3  68.1 % females;  48.8% response; | 400-m service area, based on road networks;  GIS-based walkability score  residential density, street connectivity, and land-use mix measures | within a 10 to 15-min walk;  Self-reported via NEWS-A  all subscales | self-reported via CHAMPS- questionnaire;  frequency and duration of weekly minutes of walking leisurely for exercise or pleasure, walking fast or briskly for exercise, and walking to do errands |  | *objective determinants were mainly used to model the NE or Walkability (high walkable/ low walkable areas). Not for a detailed comparison.* |
| **approach: combination** | | | | | |  |
| ***Ding et al., 2014*** (24);  SNQSL  Seattle-King County, WA, and Baltimore-Washington, DC, USA, urban  observational | n = 880;  age: mean 75  56 % females;  24.5% response;  Stratification: walkability, SES | 500m street network-buffer  GIS-based walkability index  net residential density, land-use mix, retail floor area ratio, and intersection density, access to parks &public open space, number of parks & nearby locations for recreational PA | 15-20min walk from home;  Self-reported via  older adult-modified version of NEWS  residential density, land use mix-diversity , land use mix-access, street connectivity , walking-bicycling infrastructures , aesthetics , traffic safety , pedestrian safety structures, personal safety | *self-report* via CHAMPS-questionnaire &*objective monitoring* via accelerometer (ACTi Graph)  PA and self-reported transport and leisure walking (MVPA)  moderator: driving status |  | *objective and perceived NE characteristics were overserved according to their potential different influences on PA* |
| ***Fisher et al., 2004*** (25);  56 neighborhoods in Portland, OR, USA  cross-sectional observational study | n = 582;  age: mean 73.99, SD 6.25;  68.6 % females;  30.5% response; | neighborhood defıned by the city's Office of Neighborhood Involvement  assessment with direct observations & census data aggregated to the level of the neighborhood, e.g. on parks and trails  senior population density, and facilities (e.g. parks, trails) per neighborhood acre, income, White composition | 15-20min walk from home;  Self-reported via items from NEWS  ratio of perceived problems (gangs, graffiti, violent crime, vandalism, burglary, abandoned or boarded-up, buildings, and alcohol or drug use), perceptions of neighborhood safety | self-reported  mean neighborhood walking-activity score  social cohesion |  | *objective and perceived NE characteristics were overserved according to their potential different influences on PA.* |
| ***Gauvin et al., 2012*** (26);  VoisiNuAge Study (2003-2008)  Montreal, Laval, and Sherbrooke in the province of Québec, Canada, urban  longitudinal, observational study | n = 521;  age: mean 74.7; SD 4.1  53.4 % females;  Stratification: quartiles of proximity | quartiles of proximity (first:650 m- forth: 1.9 km)  MEGAPHONE database (validated) GIS derived measures  proximity (shortest road network distance) of average distance - local services and amenities with the home | 5min walk from home  Self-reported  quality of walking environment and transportation services, user-friendliness of the walking environment, access to overall neighborhood services and amenities, accessibility to key resources for older adults | self-reported via  Physical Activity Scale for Seniors (PASE)  frequency of Walking |  | *objective and perceived NE characteristics were overserved according to their potential different influences on PA.* |
| ***Gómez et al., 2010*** (27);  2007  Fifty neighborhoods in Bogota, Columbia, urban  cross-sectional | n = 1966;  age: mean 70.7; SD 7.7  62.5 % females;  67.8% response;  Stratification: SES, urban characteristics, and bounded by urban or natural limits | as-the-crow-flies buffer of 500 m around the centroid of each NE  GIS derived measures  public park density, street connectivity, presence of a Ciclovía corridor, number of TransMilenio stations, land use mix | self-reported (interviews)  safety from traffıc, satisfaction with the quality of sidewalks | self-reported via  IPAQ short  Walking for leisure & utilitarian purposes (having walked for at least 60 min& having walked for at least 150 min during a usual week). |  | *objective and perceived NE characteristics were overserved according to their potential different influences on PA.* |
| ***Hall and McAuley, 2010*** (28);  2010  Illinois, USA  cross-sectional | n = 128;  age: mean: 69.6  100 % females; | Circular buffer of 1 km*  GIS derived measures  presence of schools, recreational areas (e.g. golf courses, recreation centers), parks, walking paths and exercise/gym facilities | *10min walk from home^∆;^*  self-reported via NEWS  residential density, land use diversity access to services, street connectivity, walking/ cycling areas, aesthetics, pedestrian safety from traffic, safety from crime and overall neighborhood satisfaction | objective via accelerometer  Daily step count over 7 days;  self-efficacy, social support, functional limitations |  | *objective and perceived NE characteristics were overserved according to their potential different influences on PA.* |
| ***Li et al., 2005*** (29);  2005  56 city defined neighborhoods in Portland, Oregon, USA  cross-sectional | n = 577;  age: mean 74, SD 6.3;  64 % females;  31% response;  Stratification: neighborhood/residential level | city defined  GIS derived measures  number of residential households, number of places of employment, number of street intersections, total green and open spaces for recreation, areas for recreation such as playgrounds (in acres). | self-reported  proximity to local recreational facilities, safety for walking, ( safety from traffic, number of nearby recreational facilities | self-reported  engaging in walking, strolling, other physical activities  perceptions of proximity |  | *objective and perceived NE characteristics were overserved according to their potential different influences on PA.* |
| ***Mowen et al., 2007*** (30);  2007  Cuyahoga County, Ohio (e.g., Greater Cleveland), USA  cross-sectional | n = 1515;  age: mean 67.4, SD 9,  66 % females;  45% response; | walking distance from home  GIS derived measures  geocoded proximity to a public park from residents home to the nearest park | walking distance from home  self-reported  park within walking distance home (0 = no, 1 = yes). | self-reported  activity level (sedentary, moderate, or active) within an average day  mediators: park visitation, daily PA | . | *objective and perceived NE characteristics were overserved according to their potential different influences on PA.* |
| ***Nathan et al., 2014 (31);***  2014  Residents in Perth, Australia  cross-sectional | n = 323;  age: mean 76.9., SD 7.3,  68.1 % females;  46 % response;  Stratification: village characteristics | 400-m service area, based on road networks,  GIS derived measures, walkability score  Distance to local services, distance to entertainment facility, distance to public transport, Distance to public recreation area, Traffic-volume exposure, slope, Walkability score | within a 10 to 15-min walk  self-reported via NEWS-A  access to services, proximate destinations, infrastructure for walking, aesthetics, safety from crime, safety from traffic, physical barriers, amenities, recreational facilities | *self-reported* via CHAMPS- questionnaire +*objectively* measured using accelerometers  frequency and duration of weekly minutes of walking leisurely for exercise or pleasure, walking fast or briskly for exercise, and walking to do errands + MVPA |  | *objective and perceived NE characteristics were overserved according to their potential different influences on PA.* |
| ***Nyunt et al., 2015*** (32);  Singapore Longitudinal Aging Study, Wave 2 (2011-2012)  South Central region of Singapore, urban  cross-sectional | n = 402;  age: mean 69.13, SD 8.53  60.7 % females; | 500m street network-buffer  GIS-derived walkability index & accessibility index, analyses of archival maps  street connectivity, residential density, land use mix, public park density, for walkability index: residential lot coverage, street density and land-use mix | self-reported via modified version of NEWS  residential density, land use mix—diversity, street connectivity, land use mix—access, infrastructure, aesthetics, traffic safety, safety from crime | self-reported  frequency of walking for Transportation |  | *objective and perceived NE characteristics were overserved according to their potential different influences on PA.* |
| ***Satariano et al., 2010*** (33);  Healthy Aging Research Network (HAN) Walking Study (2010)  Alameda County CA, Cook County IL, Allegheny County PA, and Wake and Durham counties NC, USA  cross-sectional | n = 884;  age: 65-74: 51%, ≥75  77.6 % females;  Stratification: counties | a 400-meter buffer (radial distance) *  GIS derived measures  number and types of common destinations (e.g., businesses) and, at the census tract level, street connectivity) and housing unit density. | *10min walk from home^∆;^*  self-reported via NEWS-A  all subscales: perceived residential density, land-use mix diversity, land-use mix access, street connectivity, walking/cycling facilities, aesthetics, pedestrian/automobile traffıc safety, and crime safety. | self-reported  time spent walking in a typical week ( <150 vs ≥ 150 minutes per week).  currently driver or access to driver, primary type of buildings in neighborhood |  | *objective and perceived NE characteristics were overserved according to their potential different influences on PA.* |
| ***Strath et al., 2012*** (34)  (05/2005-01/2007)  Wisconsin, USA, urban, four neighborhoods  observational study | n = 148;  age mean 64.3; SD 8.4  79.7 % females;  Stratification: high- or low walkable characteristics | 200 m network buffers *  Brownson Community Audit Tool-Analytic Version (CAT-AV)  number and variety of land uses, transportation infrastructure quality, aesthetic quality | *10min walk from home^∆;^*  self-reported via NEWS  subscales: residential density; proximity to nonresidential land uses; ease of access to nonresidential uses; street connectivity; walking/cycling facilities; aesthetics,; pedestrian safety; crime, general neighborhood satisfaction | Objective via accelerometer  total PA: MVPA |  | *objective and perceived NE characteristics were overserved according to their potential different influences on PA.* |
| ***Mathis et al., 2017*** (35)  Speak to Your Health! Community survey  (2009)  Flint, Michigan, USA, urban  cross-sectional | n = 217;  age: 65 - 91  70 % females;  Stratification: high- or low walkable characteristics | a person’s immediate residential environment, census tract  measurement of the environment via 4 Items (data: census tract, routine government database and FBI)  availability of a park , a library, grocery store in the neighborhood, and crime indices. | self-reported  crime rate compared to other neighborhoods, victim of a crime, participation in neighborhood activities | self-reported  days per week engaged in moderate physical activity, such as brisk walking, bicycling, vacuuming, gardening, for at least 10 min at a time--> activity Level |  | *objective and perceived NE characteristics were overserved according to their potential different influences on PA.* |

*notes: *around each participant’s home; ^∆^according to NEWS or PANES*

***Abbreviations:*** *ALPHA: Assessing Levels of Physical Activity and fitness at population level, BEPAS: Belgian Environmental Physical Activity Study , CHAMPS: Child Health and Mortality Prevention Surveillance, EPAQ: Electronic Personal Assessment Questionnaire, EPIC:  European Prospective Investigation into Cancer and Nutrition, GIS: Geographic Information System, MVPA: Moderate-to-Vigorous Physical Activity, NE: Neighborhood Environment, NEWS: Neighborhood Environment Walkability Scale, NEWS-A: Neighborhood Environment Walkability Scale - abbreviated version, NPAQ: Neighborhood Physical Activity Questionnaire , IPAQ: International Physical Activity Questionnaire, MARSHAL: MESA: Medical, Epidemiologic, and Social aspects of Aging, OSM: Open Street Maps, PA: Physical activity, PANES:* *Physical Activity Neighborhood Environment Survey, PASE: Physical Activity Scale for Seniors , SHAPE:* *Survey of the Health of All the Population and Environment, SNAP: Supplemental Nutrition Assistance Program, SNQL: Neighborhood Quality of Life Study for Seniors, SPOTLIGHT: Sustainable prevention of obesity through integrated strategies*

1. Arvidsson D, Kawakami N, Ohlsson H, Sundquist K. Physical activity and concordance between objective and perceived walkability. Med Sci Sports Exerc. 2012;44(2):280-7.

2. Compernolle S, De Cocker K, Roda C, Oppert JM, Mackenbach JD, Lakerveld J, et al. Physical Environmental Correlates of Domain-Specific Sedentary Behaviours across Five European Regions (the SPOTLIGHT Project). PLoS One. 2016;11(10):e0164812.

3. Dadvand P, Bartoll X, Basagana X, Dalmau-Bueno A, Martinez D, Ambros A, et al. Green spaces and General Health: Roles of mental health status, social support, and physical activity. Environ Int. 2016;91:161-7.

4. Hajna S, Ross NA, Joseph L, Harper S, Dasgupta K. Neighbourhood Walkability and Daily Steps in Adults with Type 2 Diabetes. PLoS One. 2016;11(3):e0151544.

5. Michael Y, Beard T, Choi D, Farquhar S, Carlson N. Measuring the influence of built neighborhood environments on walking in older adults. J Aging Phys Act. 2006;14(3):302-12.

6. Wu YT, Jones NR, van Sluijs EM, Griffin SJ, Wareham NJ, Jones AP. Perceived and Objectively Measured Environmental Correlates of Domain-Specific Physical Activity in Older English Adults. J Aging Phys Act. 2016;24(4):599-616.

7. Duncan M, Mummery K. Psychosocial and environmental factors associated with physical activity among city dwellers in regional Queensland. Prev Med. 2005;40(4):363-72.

8. Forjuoh SN, Ory MG, Won J, Towne SD, Jr., Wang S, Lee C. Determinants of Walking among Middle-Aged and Older Overweight and Obese Adults: Sociodemographic, Health, and Built Environmental Factors. J Obes. 2017;2017:9565430.

9. Hanibuchi T, Nakaya T, Yonejima M, Honjo K. Perceived and Objective Measures of Neighborhood Walkability and Physical Activity among Adults in Japan: A Multilevel Analysis of a Nationally Representative Sample. International Journal of Environmental Research and Public Health. 2015;12(10):13350.

10. Hu SC, Huang NC, Lin YT, Kung SF, Lin LL. Associations between physical facilities and organizational participation and levels of physical activity of adults in Tainan, Taiwan. J Phys Act Health. 2013;10(8):1109-18.

11. Piro FN, Noss O, Claussen B. Physical activity among elderly people in a city population: the influence of neighbourhood level violence and self perceived safety. J Epidemiol Community Health. 2006;60(7):626-32.

12. Towne SD, Jr., Won J, Lee S, Ory MG, Forjuoh SN, Wang S, et al. Using Walk Score and Neighborhood Perceptions to Assess Walking Among Middle-Aged and Older Adults. J Community Health. 2016;41(5):977-88.

13. Trinh L, Larsen K, Faulkner GE, Plotnikoff RC, Rhodes RE, North S, et al. Social-ecological correlates of physical activity in kidney cancer survivors. J Cancer Surviv. 2016;10(1):164-75.

14. Bracy NL, Millstein RA, Carlson JA, Conway TL, Sallis JF, Saelens BE, et al. Is the relationship between the built environment and physical activity moderated by perceptions of crime and safety? Int J Behav Nutr Phys Act. 2014;11(1):24.

15. King D. Neighborhood and Individual Factors in Activity in Older Adults: Results From the Neighborhood and Senior Health Study. Journal of Aging and Physical Activity. 2008;16(2):144-70.

16. Nagel CL, Carlson NE, Bosworth M, Michael YL. The relation between neighborhood built environment and walking activity among older adults. Am J Epidemiol. 2008;168(4):461-8.

17. Ng TP, Nyunt MSZ, Shuvo FK, Eng JY, Yap KB, Hee LM, et al. The Neighborhood Built Environment and Cognitive Function of Older Persons: Results from the Singapore Longitudinal Ageing Study. Gerontology. 2018;64(2):149-56.

18. Orstad SL, McDonough MH, James P, Klenosky DB, Laden F, Mattson M, et al. Neighborhood walkability and physical activity among older women: Tests of mediation by environmental perceptions and moderation by depressive symptoms. Prev Med. 2018;116:60-7.

19. Troped PJ, Tamura K, McDonough MH, Starnes HA, James P, Ben-Joseph E, et al. Direct and Indirect Associations Between the Built Environment and Leisure and Utilitarian Walking in Older Women. Annals of Behavioral Medicine. 2017;51(2):282-91.

20. Van Holle V, Van Cauwenberg J, Gheysen F, Van Dyck D, Deforche B, Van de Weghe N, et al. The Association between Belgian Older Adults' Physical Functioning and Physical Activity: What Is the Moderating Role of the Physical Environment? PLoS One. 2016;11(2):e0148398.

21. Bodeker M. Walking and Walkability in Pre-Set and Self-Defined Neighborhoods: A Mental Mapping Study in Older Adults. Int J Environ Res Public Health. 2018;15(7).

22. Lee JS, Kawakubo K, Kohri S, Tsujii H, Mori K, Akabayashi A. Association between residents' perception of the neighborhood's environments and walking time in objectively different regions. Environ Health Prev Med. 2007;12(1):3-10.

23. Nathan A, Wood L, Giles-Corti B. Perceptions of the built environment and associations with walking among retirement village residents. Environment and Behavior. 2012;46(1):46-69.

24. Ding D, Sallis JF, Norman GJ, Frank LD, Saelens BE, Kerr J, et al. Neighborhood environment and physical activity among older adults: Do the relationships differ by driving status? Journal of Aging and Physical Activity. 2014;22(3):421-31.

25. Fisher KJ, Li F, Michael Y, Cleveland M. Neighborhood-level influences on physical activity among older adults: a multilevel analysis. J Aging Phys Act. 2004;12(1):45-63.

26. Gauvin L, Richard L, Kestens Y, Shatenstein B, Daniel M, Moore SD, et al. Living in a well-serviced urban area is associated with maintenance of frequent walking among seniors in the VoisiNuAge study. J Gerontol B Psychol Sci Soc Sci. 2012;67(1):76-88.

27. Gómez LF, Parra DC, Buchner D, Brownson RC, Sarmiento OL, Pinzón JD, et al. Built environment attributes and walking patterns among the elderly population in Bogotá. American journal of preventive medicine. 2010;38(6):592-9.

28. Hall KS, McAuley E. Individual, social environmental and physical environmental barriers to achieving 10 000 steps per day among older women. Health education research. 2010;25(3):478-88.

29. Li F, Fisher KJ, Brownson RC, Bosworth M. Multilevel modelling of built environment characteristics related to neighbourhood walking activity in older adults. Journal of Epidemiology & Community Health. 2005;59(7):558-64.

30. Mowen A, Orsega-Smith E, Payne L, Ainsworth B, Godbey G. The role of park proximity and social support in shaping park visitation, physical activity, and perceived health among older adults. Journal of Physical Activity and Health. 2007;4(2):167-79.

31. Nathan A, Wood L, Giles-Corti B. Exploring socioecological correlates of active living in retirement village residents. Journal of aging and physical activity. 2014;22(1):1-15.

32. Nyunt MS, Shuvo FK, Eng JY, Yap KB, Scherer S, Hee LM, et al. Objective and subjective measures of neighborhood environment (NE): relationships with transportation physical activity among older persons. Int J Behav Nutr Phys Act. 2015;12:108.

33. Satariano WA, Ivey SL, Kurtovich E, Kealey M, Hubbard AE, Bayles CM, et al. Lower-body function, neighborhoods, and walking in an older population. American journal of preventive medicine. 2010;38(4):419-28.

34. Strath SJ, Greenwald MJ, Isaacs R, Hart TL, Lenz EK, Dondzila CJ, et al. Measured and perceived environmental characteristics are related to accelerometer defined physical activity in older adults. Int J Behav Nutr Phys Act. 2012;9:40.

35. Mathis AL, Rooks RN, Tawk RH, Kruger DJ. Neighborhood Influences and BMI in Urban Older Adults. J Appl Gerontol. 2017;36(6):692-708.
