# Supplementary material for: Measuring the association of objective and perceived neighborhood environment with physical activity in older adults: challenges and implications from a systematic review
Source: Int J Health Geogr. 2020 Nov 9;19:47. doi: 10.1186/s12942-020-00243-z (PMC7654613; doi:10.1186/s12942-020-00243-z)
Supplement: Supplementary file 6 — Additional file 6. Results of the methodological quality assessment of the individual studies. [file 12942_2020_243_MOESM6_ESM.docx]

**Additional file 6: Results of the methodological quality assessment of the individual studies**

|  | **Representativeness** | | **Confounder** | | **Data collection** | | | **Results** |
| --- | --- | --- | --- | --- | --- | --- | --- | --- |
| **Author, year** | **Study area** | **Selection** | **on individual level** | **on NE level** | **Objective NE measures** | **Perceived NE measures** | **PA measures (Outcome)** | **Analytical Approach** |
|  | *Participants recruitment stratified by environmental attributes* | *Response rate > 60% or sample representative of the population* | *Controlled for/stratified by age, sex, SES, health status, etc.* | | *Measures are described, valid or well established in the field* | | | *Analyses conducted and presented correctly*  *(e.g., confidence intervals, p-values indicated)* |
| **Approach: direct comparison** | | | | | | | | |
| Arvidsson et al., 2012 | ⚫ | ⚫ | ⚫ | ⚫ | ⚫ | ⚫ | ⚫ | ⚫ |
| Compernolle et al., 2016 | ⚫ | ⚫ | ⚫ | ⚫ | ⚫ | ⚫ | ⚫ | ⚫ |
| Dadvand et al., 2016 | ⚫ | ⚫ | ⚫ | ⚫ | ⚫ | ⚫ | ⚫ | ⚫ |
| Hajna et al., 2016 | ⚫ | ⚫ | ⚫ | ⚫ | ⚫ | ⚫ | ⚫ | ⚫ |
| Michael et al., 2006 | ⚫ | ⚫ | ⚫ | ⚫ | ⚫ | ⚫ | ⚫ | ⚫ |
| Wu et al., 2016 | ⚫ | ⚫ | ⚫ | ⚫ | ⚫ | ⚫ | ⚫ | ⚫ |
| **Approach: indirect comparison** | | | | | | | | |
| Duncan &Mummery, 2005 | ⚫ | ⚫ | ⚫ | ⚫ | ⚫ | ⚫ | ⚫ | ⚫ |
| Forjuoh et al., 2017 | ⚫ | ⚫ | ⚫ | ⚫ | ⚫ | ⚫ | ⚫ | ⚫ |
| Hanibuchi et al., 2015 | ⚫ | ⚫ | ⚫ | ⚫ | ⚫ | ⚫ | ⚫ | ⚫ |
| Hu et al., 2013 | ⚫ | ⚫ | ⚫ | ⚫ | ⚫ | ⚫ | ⚫ | ⚫ |
| Piro et al., 2006 | ⚫ | ⚫ | ⚫ | ⚫ | ⚫ | ⚫ | ⚫ | ⚫ |
| Towne et al., 2016 | ⚫ | ⚫ | ⚫ | ⚫ | ⚫ | ⚫ | ⚫ | ⚫ |
| Trinh et al., 2016 | ⚫ | ⚫ | ⚫ | ⚫ | ⚫ | ⚫ | ⚫ | ⚫ |
| **Approach: interaction/moderation** | | | | | | | | |
| Bracy et al., 2014 | ⚫ | ⚫ | ⚫ | ⚫ | ⚫ | ⚫ | ⚫ | ⚫ |
| King, 2008 | ⚫ | ⚫ | ⚫ | ⚫ | ⚫ | ⚫ | ⚫ | ⚫ |
| Nagel et al., 2008 | ⚫ | ⚫ | ⚫ | ⚫ | ⚫ | ⚫ | ⚫ | ⚫ |
| Ng et al., 2018 | ⚫ | ⚫ | ⚫ | ⚫ | ⚫ | ⚫ | ⚫ | ⚫ |
| Orstad et al., 2018 | ⚫ | ⚫ | ⚫ | ⚫ | ⚫ | ⚫ | ⚫ | ⚫ |
| Troped et al., 2017 | ⚫ | ⚫ | ⚫ | ⚫ | ⚫ | ⚫ | ⚫ | ⚫ |
| Van Holle et al., 2016 | ⚫ | ⚫ | ⚫ | ⚫ | ⚫ | ⚫ | ⚫ | ⚫ |
|  | **Representativeness** | | **Confounder** | | **Data collection** | | | **Results** |
| **Author, year** | **Study area** | **Selection** | **on individual level** | **on NE level** | **Objective NE measures** | **Perceived NE measures** | **PA measures (outcome)** | **Analytical Approach** |
|  | *Participants recruitment stratified by environmental attributes* | *Response rate > 60% or sample representative of the population* | *Controlled for/stratified by age, sex, SES, health status, etc.* | | *Measures are described, valid or well established in the field* | | | *Analyses conducted and presented correctly*  *(e.g., confidence intervals, p-values indicated))* |
| **Approach: modeling** | | | | | | | | |
| Bodeker, 2018 | ⚫ | ⚫ | ⚫ | ⚫ | ⚫ | ⚫ | ⚫ | ⚫ |
| Lee et al., 2007 | ⚫ | ⚫ | ⚫ | ⚫ | ⚫ | ⚫ | ⚫ | ⚫ |
| Nathan et al., 2012 | ⚫ | ⚫ | ⚫ | ⚫ | ⚫ | ⚫ | ⚫ | ⚫ |
| **Approach: combination** | | | | | | | | |
| Ding et al., 2014 | ⚫ | ⚫ | ⚫ | ⚫ | ⚫ | ⚫ | ⚫ | ⚫ |
| Fisher et al., 2004 | ⚫ | ⚫ | ⚫ | ⚫ | ⚫ | ⚫ | ⚫ | ⚫ |
| Gauvin et al., 2012 | ⚫ | ⚫ | ⚫ | ⚫ | ⚫ | ⚫ | ⚫ | ⚫ |
| Gómez et al., 2010 | ⚫ | ⚫ | ⚫ | ⚫ | ⚫ | ⚫ | ⚫ | ⚫ |
| Hall and McAuley, 2010 | ⚫ | ⚫ | ⚫ | ⚫ | ⚫ | ⚫ | ⚫ | ⚫ |
| Li et al., 2005 | ⚫ | ⚫ | ⚫ | ⚫ | ⚫ | ⚫ | ⚫ | ⚫ |
| Mowen et al., 2007 | ⚫ | ⚫ | ⚫ | ⚫ | ⚫ | ⚫ | ⚫ | ⚫ |
| Nyunt et al., 2015 | ⚫ | ⚫ | ⚫ | ⚫ | ⚫ | ⚫ | ⚫ | ⚫ |
| Satariano et al., 2010 | ⚫ | ⚫ | ⚫ | ⚫ | ⚫ | ⚫ | ⚫ | ⚫ |
| Strath et al., 2012 | ⚫ | ⚫ | ⚫ | ⚫ | ⚫ | ⚫ | ⚫ | ⚫ |
| Mathis et al., 2017 | ⚫ | ⚫ | ⚫ | ⚫ | ⚫ | ⚫ | ⚫ | ⚫ |

⚫ *yes* ⚫ *no* ⚫ *N.A or described, but not yet validated or established in the field (e.g. because of adaption)*

***Abbreviations:*** *NE: Neighborhood Environment, PA: Physical activity*
